# Predictability of Periodontal Surgical Guides Manufactured by Digital Flow in Clinical Crown Lengthening. A Randomized Trial

**DOI:** 10.1111/jerd.13487

**Published:** 2025-05-27

**Authors:** Victor Gonçalves, Carolina Mendonça de Almeida Malzoni, José Cleveilton Dos Santos, Guilherme José Pimentel Lopes de Oliveira, Marcelo Gonçalves, Elcio Marcantonio

**Affiliations:** ^1^ Department of Diagnosis and Surgery Universidade Estadual Paulista ‐ Unesp, School of Dentistry Araraquara Brazil; ^2^ Department of Periodontology Universidade Federal de Uberlândia (UFU), School of Dentistry at Uberlândia Uberlândia Brazil; ^3^ Post Graduation Course in Implantology Instituto Latino‐Americano de Pesquisa Odontológica (ILAPEO) Curitiba Brazil

**Keywords:** biological space, gingivectomy, osteotomy, periodontal surgical guide

## Abstract

**Objective:**

This randomized controlled trial aimed to evaluate the effectiveness of periodontal surgical guides manufactured using a digital workflow in aesthetic clinical crown lengthening procedures.

**Methods:**

Twenty patients with altered passive eruption in the anterior maxilla requiring clinical crown lengthening were randomly divided into two groups: control group—free hand and test group—periodontal surgical guides. All patients underwent CBCT and intraoral digital scanning (IOS) examinations before the surgical procedure. Virtual planning was carried out in both groups, measuring the height of the gingival tissue to be removed. However, the surgical guides were only printed in the test group. IOS was performed on all patients 6 and 12 months after the surgical procedure to verify the predictability of the surgical technique in relation to the initial plan. Patients also answered a visual analog scale to measure postoperative pain.

**Results:**

The comparative analysis between the test and control groups showed no statistical difference in the amount of tissue planned to be removed, and the result obtained after 6 months and 1 year (Control group: median of −0.09 to 0.29 mm in 6 months and 0.00 to 0.55 mm in 12 months. Test group: median of −0.05 to 0.70 mm in 6 months and 0.00 to 0.81 mm in 12 months). No differences were noticed between the techniques tested concerning the positioning of the gingival margin in the 6‐ or 12‐month periods in relation to the marginal tissues observed at the baseline period (*p* > 0.05). The level of pain presented by patients was low in both groups, with reports of minimal discomfort after the 15‐day follow‐up period. Patients demonstrated high satisfaction levels with the procedure regardless of the surgical technique.

**Conclusion:**

Both guided and conventional surgery techniques are effective for treating altered passive eruption. However, the painful sensation and tendency for recurrence of the coronal positioning of the marginal tissues were lower when the guided surgery technique was applied.

**Clinical Significance:**

This study will help clinicians understand which surgical technique for treating altered passive eruptions is more appropriate for their clinical case.

## 1 Introduction

Managing a gummy smile is challenging due to the patient's high aesthetic expectations [1]. Therefore, the predictability of obtaining good results is essential for developing diagnostic methods that can influence clinical management [2]. The gummy smile correction can involve several dental specialties, as the cause of this condition can vary. Lip elevator muscle hyperfunction, maxilla excessive vertical growth, short upper lip, or even altered passive eruption of the teeth are possible causes [3]. In periodontics, gummy smiles and discrepancies between the measurements of dental elements can be corrected through clinical crown enlargement. To perform this technique, it is necessary to locate the cemento‐enamel junction (CEJ) to indicate the maximum amount of gingival tissue that can be removed without causing root exposure [2, 3].

Traditionally, the location of the CEJ was ascertained through probing and the professional's tactile sensitivity, leading to risks concerning removing excess gingival tissue [2, 4, 5]. A new phase for periodontal surgery began with cone beam computed tomography (CBCT) to determine the CEJ and measure the relationship between gingival and bone tissue. The process includes obtaining the digital imaging of surgical procedures, planning on a computer, and executing the planned procedures on the day of surgery [6]. Tomographic planning of these procedures provides prior information about the necessity or not of removing bone tissue and accurately defines the measurement of the tissues that need to be removed [6, 7].

Concerning clinical crown lengthening procedures, CBCT can provide faithful measurements of the relationship between bone tissue and gingival tissue, reducing the risk of damage to adjacent anatomical structures during the surgical procedure [8, 9]. Additionally, diagnostic wax‐ups can be performed on study models [2, 10]. In this way, it is possible to allow the patients to visualize the result before they undergo the procedure and to decide whether the result is in accordance with their expectations [2, 10]. However, this handmade waxing is not predictable enough to be used as a guide [11].

With the advancement of the digital era, the wax‐up on the study model has been replaced by a virtual wax‐up based on a patient's intra‐oral scan [11]. Furthermore, through the overlap between the CBCT and the scan, a surgical guide can be printed, further contributing to the planning and precision of the surgical procedure. Using digital tools assists in planning and reducing treatment risks, as surgical guides reduce discrepancies that may occur when performing the technique [11, 12, 13]. Furthermore, using digital tools increases communication with the patient, providing a better and more faithful view of the result [12, 13]. However, the use of digital resources is still considered recent and involves high‐cost equipment.

Few studies have evaluated the use of periodontal surgical guides for crown lengthening. Recent studies found no differences in the gingival marginal stability between the two techniques [14, 15]. Therefore, the present study aims to evaluate if the predictability of clinical crown augmentation procedures in the aesthetic area using periodontal surgical guides made with digital flow is superior to performing the technique without using guides. The null hypothesis was that there would be no significant difference concerning the predictability of the positioning of the gingival margin when using or not using a periodontal surgical guide for clinical crown lengthening.

## 2 Material and Methods

This controlled parallel randomized clinical trial was submitted and approved by the Research Ethics Committee of the XXXX (XXXX, CAAE number XXXX). The study was also registered on the Brazilian Clinical Trials Registry Platform (XXXXX) (Approval date: XX/XX/XXXX). All patients read and signed the informed consent form before being included in this trial. The current study followed the ethical statements of the Declaration of Helsinki. Furthermore, the study was conducted according to the CONSORT Guidelines.

### 2.1 Eligibility Criteria

To be included in this study, the patients needed to complain of a gummy smile due to altered passive eruption, requiring osteotomy to reestablish biological space in the region of teeth 14 to 24. The patients' age was between 18 and 40 years of both genders. Patients had to be systemically healthy and not use medications that alter the inflammatory process or bone metabolism.

Patients who had undergone radiotherapy treatment in the head and neck region, immunosuppressed patients, patients who were taking or had used anti‐resorptive medications or drugs that alter bone metabolism, patients with poor oral hygiene, pregnant or lactating women, patients with uncontrolled diabetes, smokers, and patients with unrealistic expectations were excluded. Despite the keratinized gingiva width, bleeding on probing, and periodontal health not being considered in the exclusion criteria, a general periodontal evaluation was performed in all study patients, and no abnormalities were found.

### 2.2 Study Design and Sample Size Calculation

Twenty patients with altered passive eruption in the anterior maxilla (14–24 teeth) were randomly allocated into two groups: Control group—The clinical crown lengthening procedures were performed without the use of periodontal surgical guides; Test group—The clinical crown lengthening procedures were performed using periodontal surgical guides. Randomization was performed at the patient level using a random allocation sequence generated by software, with a random ratio of 1:1 (www.randomizer.org).

The primary variable of this study was the stability of the gingival margin after surgery. The sample size was calculated based on a previous study and the clinical relevance of the present study's primary parameter. Considering a type I error of 5% and study power of 75% to detect a minimal mean difference between groups, with a standard deviation of 0.66 mm [14], and taking into account that the 1 mm difference in the relationship between the margin and the cementoenamel junction is clinically relevant in crown augmentation surgeries, the minimum number of patients to be treated in this study was estimated at 10 patients per group.

### 2.3 Initial Planning

**FIGURE 1:**
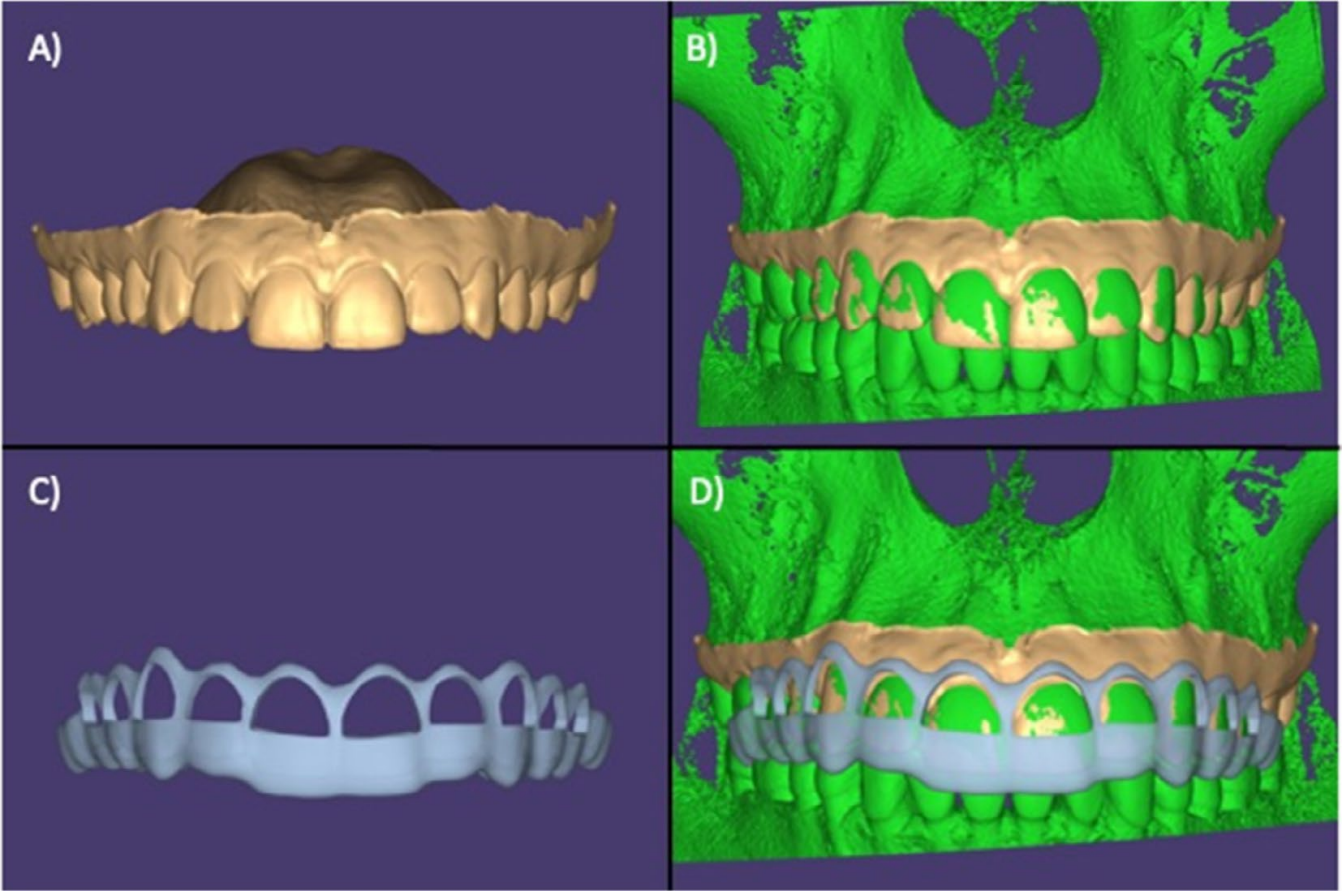
Image of the virtual planning used to create the periodontal guide with Exocad Version DentalDB 2.3 Matera software (Exocad America Inc.). (A) Image from the intraoral scan file (STL). (B) Overlay of the STL with the tomographic image file (DICOM). (C) Image of the periodontal guide design. (D) Overlay of the periodontal guide in DICOM and STL files.

**FIGURE 2:**
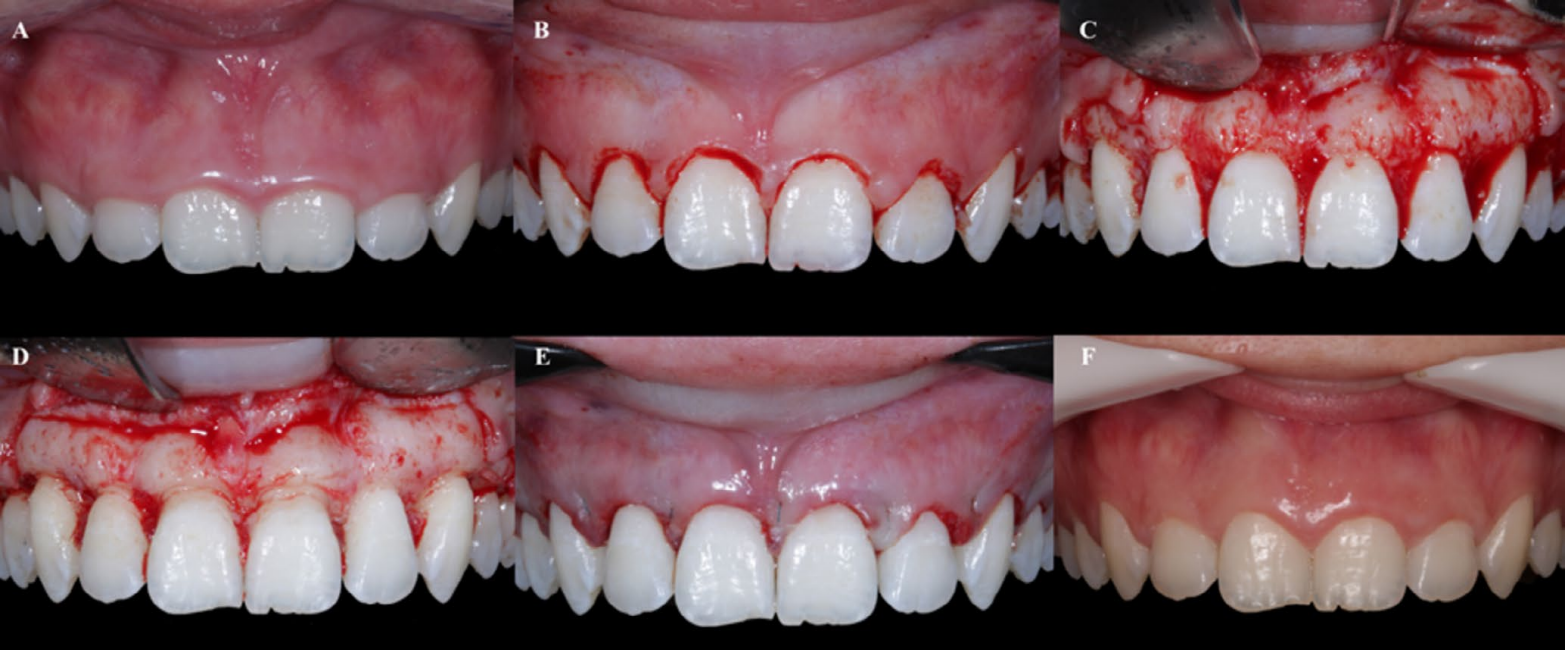
Clinical sequence of the clinical crown lengthening procedure in the control group without the use of the periodontal guide. (A) Initial clinical image. (B) Clinical aspect after the “free hand” incision. (C) Detachment of the mucoperiosteal flap. (D) Aspect after the osteotomy procedure. (E) Immediate postoperative condition. (F) Clinical outcome after 1‐year of follow‐up.

**FIGURE 3:**
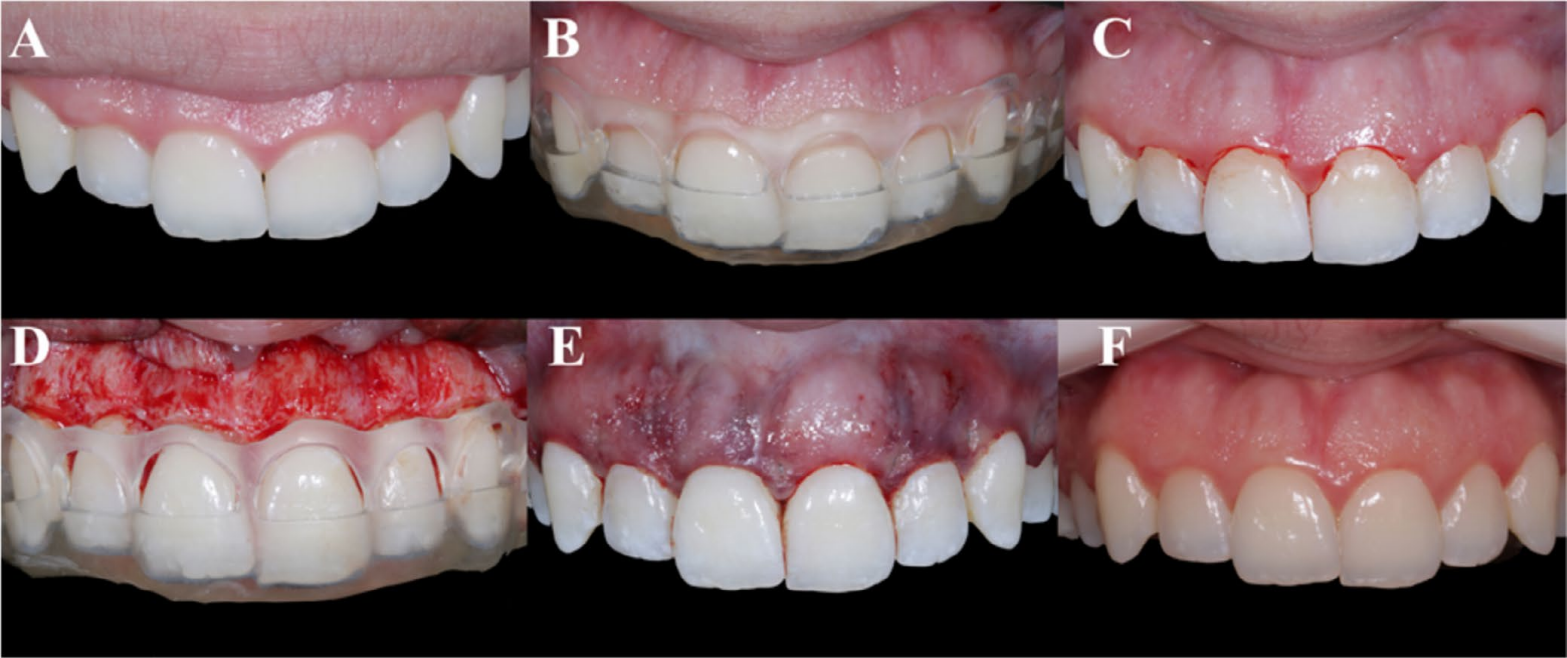
Clinical sequence of the clinical crown lengthening procedure in the test group using the periodontal guide. (A) Initial clinical image. (B) Periodontal guide in position. (C) Incision design using the periodontal guide. (D) Mucoperiosteal detachment of the flap and osteotomy using the periodontal guide. (E) Immediate postoperative period. (F) Clinical outcome after 1‐year of follow‐up.

The selected patients were submitted to initial cone beam computed tomography (CBCT) exams using VeraView X800 tomography (J.Morita, Tokyo, Japan) and intra‐oral digital scanning (E1) using the iTero Element 2 scanner (Align Technology Inc., USA). Subsequently, virtual planning was carried out, with the measurement of the tissues that should be restored (Figure 1). After planning, patients were randomly divided into two groups. The control group involved traditional clinical crown lengthening procedures without periodontal surgical guides but with access to CBCT (Figure 2). In contrast, the test group involved clinical crown lengthening procedures using periodontal surgical guides (Figure 3).

### 2.4 Surgical Procedure

**FIGURE 4:**
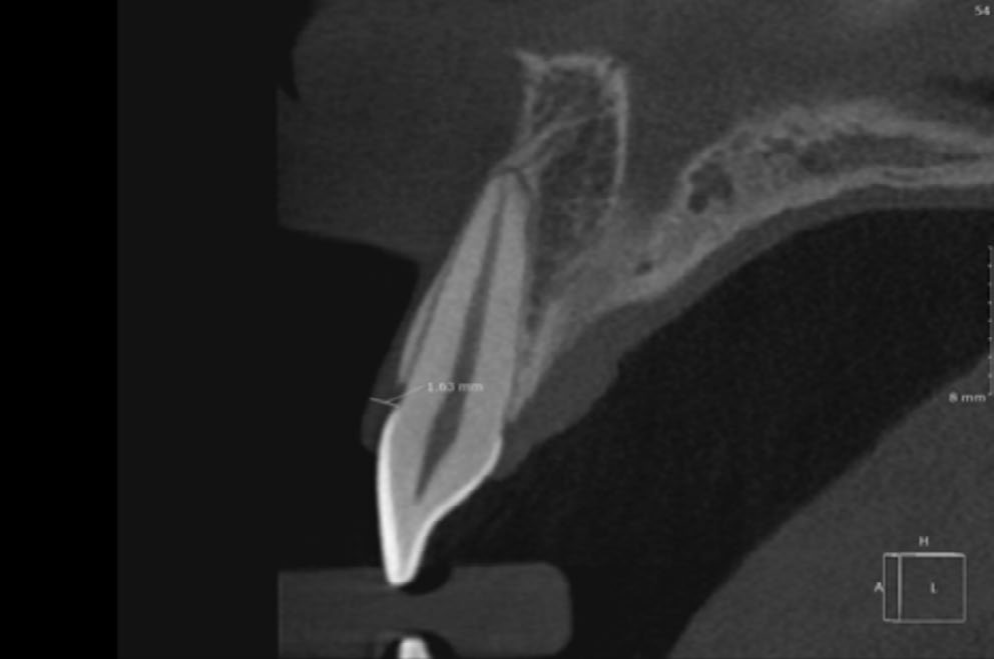
Sagittal section of the cone beam computed tomography, indicating the measurement of gingival thickness perpendicular to the cementoenamel junction.

Before the surgical procedure, the patient's gingival phenotype was analyzed using cone beam computed tomography (Figure 4). Gingival thickness > 1 mm at the cemento‐enamel junction level was considered a thick phenotype, and gingival thickness ≤ 1 mm at the cemento‐enamel junction level was considered a thin phenotype [16].

Patients rinsed their mouth with 15 mL of 0.12% chlorhexidine digluconate for 1 min. Local anesthesia (Articaine 4% 1:100.000—Nova DFL, Brazil) was performed in the upper anterior region. Afterward, in the control group, markings were made on the gingival margin, referring to the tissue height to be removed, and then an incision was made joining these markings. The gingival tissue collar was removed using a curette, and then the gingivoplasty was refined. Subsequently, the muco‐periosteal flap was detached, and the osteotomy was performed to reestablish the biological space. The amount of tissue removed during osteotomy depended on the gingival phenotype [17]. In the current study, in cases that presented a thick gingival phenotype, the bone level after the osteotomy was 3 mm below the CEJ, while in cases with a thin gingival phenotype, the bone level after the osteotomy was 2 mm below the CEJ. Finally, the root surfaces of the dental elements were smoothed with curettes, and vertical mattress sutures were performed. In the test group, the procedure was performed the same way; instead of marking the tissues, the surgical guide was used, offering the surgeon the limits of the tissue margins that should be removed and contoured.

The procedures were performed by the same experienced professional who only found out which group the patient was allocated to at the surgical procedure. The postoperative medication included the prescription of Amoxicillin 875 mg every 12 h for 7 days; Nimesulid 100 mg every 12 h for 3 days; Sodium Dipyrone 500 mg every 6 h for 3 days; and 0.12% chlorhexidine digluconate twice a day for 15 days.

### 2.5 Data Analysis

The patient's pain perception was evaluated 3, 7, 15, and 30 days after the surgical procedure, using a visual analog scale (VAS) in the form of a 10 cm horizontal line, where 0 (left side) indicates minimum satisfaction and 10 (right side) indicates maximum satisfaction. After the treatment, the patients were instructed to mark the best position to represent their general pain perception. The score was measured in centimeters from the left end of the line to the marked point.

**FIGURE 5:**
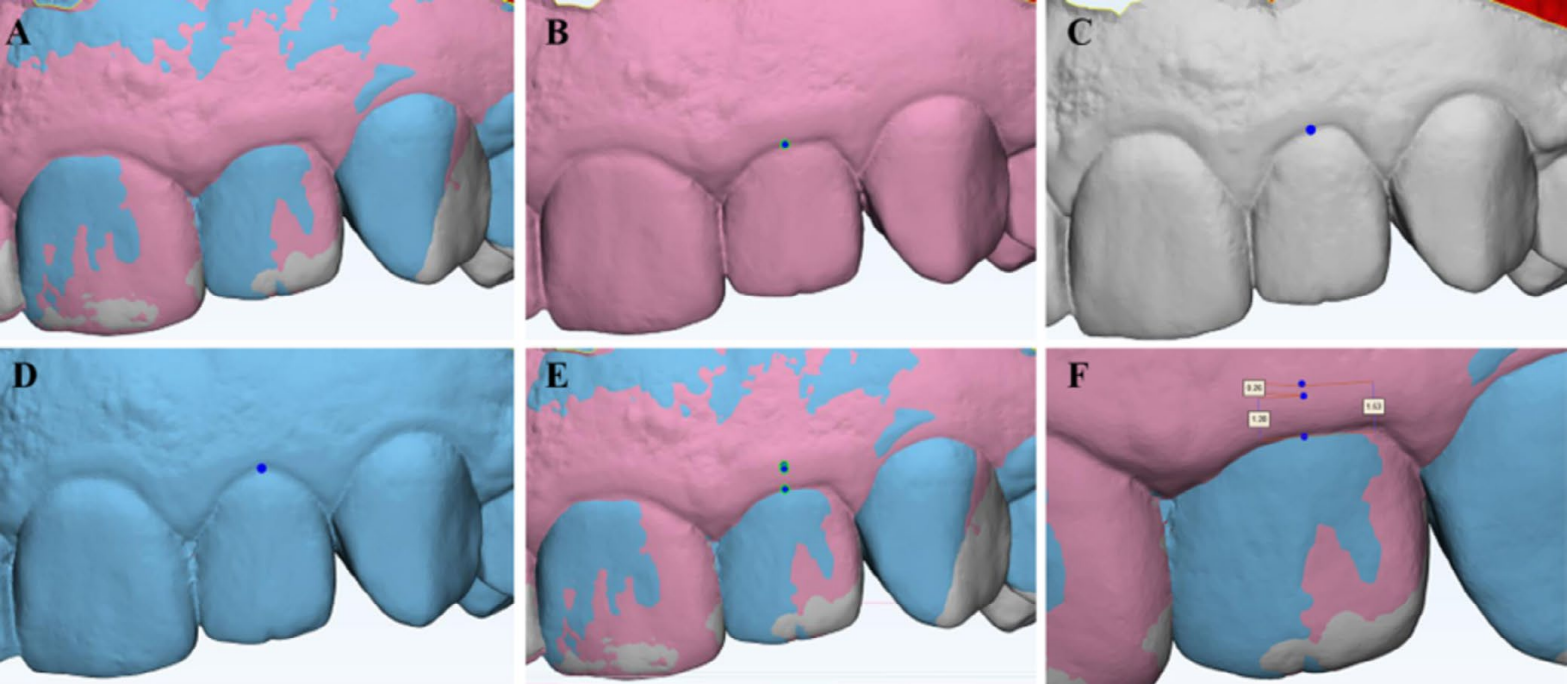
Analysis using 3‐matic software (Materialize, Leuven, Belgium). (A) Overlay of the 3 STL files (initial, after 6 months, and after 1 year). (B) Initial STL file marking the positioning of the gingival margin. (C) STL file after 6 months marking the positioning of the gingival margin. (D) STL file after 1 year marking the positioning of the gingival margin. (E) Overlay of the 3 STL files (initial, after 6 months, and after 1 year) indicating all the positions of the gingival margin at the 3 moments of analysis. (F) The 3 overlapping STL files measuring changes in gingival positioning in the STL files.

Six months after the surgical procedure, patients underwent a new intra‐oral scan (E2). This scan was superimposed on the patient's initial virtual plan (E1) using 3‐matic and Mimics software (Materialize, Leuven, Belgium) (Figure 5). The analysis was based on the best‐fit algorithm and used STL exported with the highest resolution. The difference between the initial plan and the surgical result was then analyzed and compared between the test and control groups in the regions of teeth 14 to 24.

Furthermore, 1 year after the surgical procedure, a new scan was performed (E3). It was again superimposed on the initial plan to check the procedure's predictability and on the STL of E2 to evaluate tissue stability of the gingival margin in the long term. One year after the surgical procedure, a questionnaire was sent to participants to assess their level of satisfaction with the result of the clinical crown lengthening procedure on a scale of 0 to 10, and whether the patients would recommend the surgical procedures.

### 2.6 Statistical Analysis

The study's quantitative parameters were described by median, interquartile range, and standard deviation, and the qualitative parameters by frequencies and percentages. The normality of the errors was analyzed using box plots, quantile‐quantile plots, and the Shapiro–Wilk test. These initial analyses indicated that the data did not meet the assumptions required for a parametric analysis. The comparison of the stability of clinical data on the gingival margin and the VAS Scale was compared using the Mann–Whitney test, which compares the treatment and tooth impact on these outcomes, while the evaluation within each group was performed using the Friedman test followed by the Dun Test. The results were considered significant at *p* < 0.05. All analyses were performed using the Jamovi software (https://www.jamovi.org/).

## 3 Results

**FIGURE 6:**
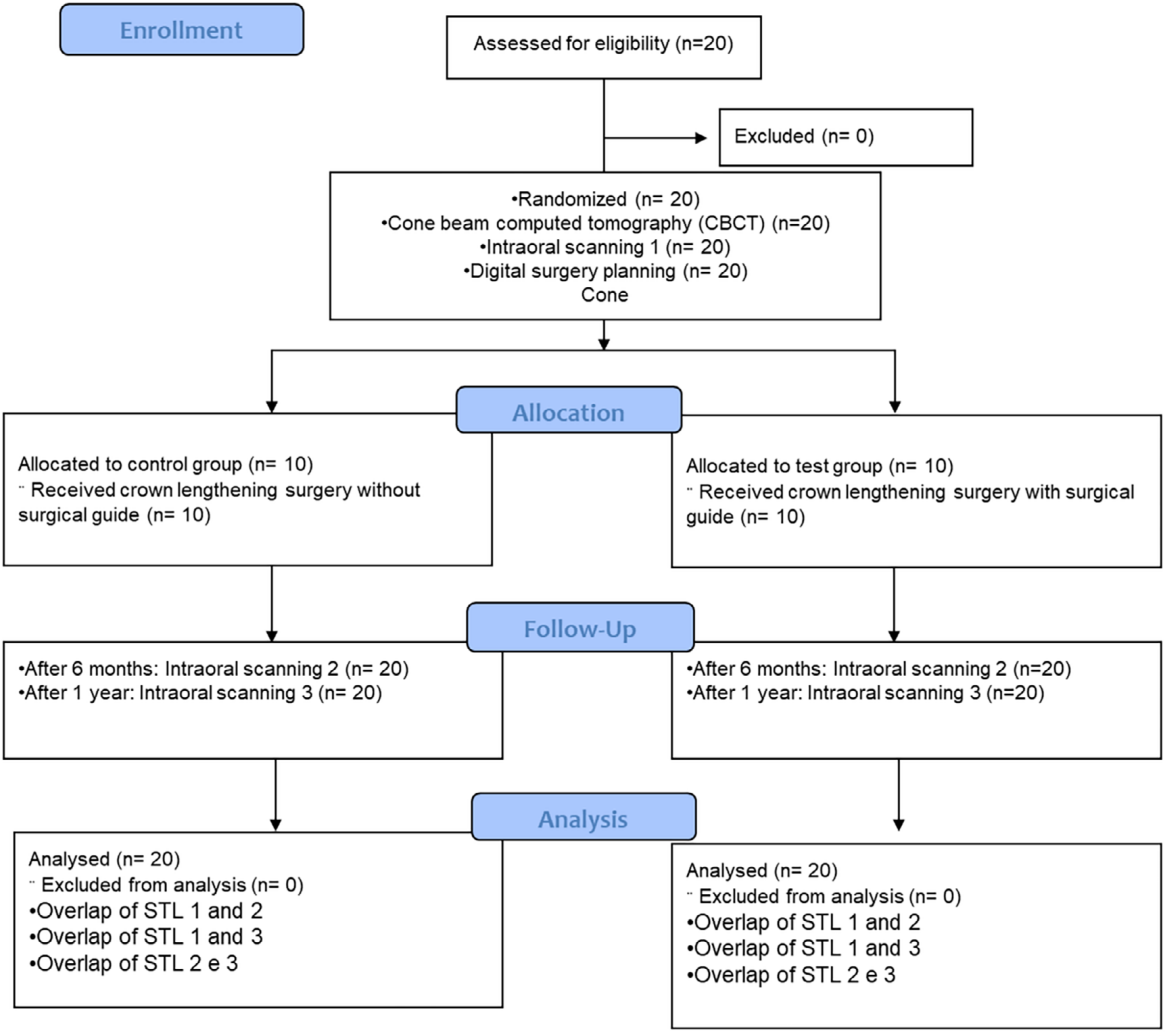
Study's flowchart.

Twenty patients (17 female and 3 male) with a mean age of 23.25 (± 3.5) years were included in this clinical study. The demographic distribution of the sample did not significantly differ between the test and control groups (Table 1). Figure 6 shows the flowchart of the different phases of the study.

**TABLE 1:** Distribution of demographic data between the test and control groups.

Parameter		Control	Test
Gender	Male	2	1
	Female	8	9
Age		23.60 ± 3.53	22.90 ± 3.57
Gingival phenotype	Thin	4	2
	Thick	6	8

### 3.1 Overlapping Analysis of STL Files

It can be observed that a lower need for gingival tissue removal on premolars compared to anterior teeth was indicated in the digital planning (Table 2). Furthermore, comparing the initial STL files with 6 and 12 months postoperatively, there was no difference in relation to the amount of gingival tissue planned to be removed and the result obtained at 6 and 12 months in all the teeth groups. All teeth evaluated showed a more coronal location of the removed gingival margin in relation to what was planned to be removed (< 0.5 mm).

**TABLE 2:** Median and interquartile range of the removal of marginal gingival tissue planned and executed.

Group	Period/Tooth	14	13	12	11	21	22	23	24
Control	Planned	0.25 (0.00; 1.00)	1.00 (0.87; 2.00)	1.50 (1.00; 2.00)	1.50 (1.00; 2.12)	1.25 (1.00; 2.00)	1.25 (1.00; 2.00)	1.00 (0.75; 2.00)	0.25 (0.00; 1.00)
	6 months	0.38 (0.13; 0.60)	1.01 (0.33; 1.40)	1.55 (0.68; 1.93)	1.42 (0.67; 1.72)	1.42 (0.97; 1.87)	1.24 (0.84; 1.62)	1.13 (0.23; 1.40)	0.32 (0.12; 0.77)
	12 months	0.41 (0.07; 0.52)	0.76 (0.38; 1.19)	1.19 (0.55; 1.60)	1.23 (0.59; 1.33)	1.05 (0.73; 1.55)	0.94 (0.70; 1.34)	0.84 (0.59; 1.22)	0.05 (0.00; 0.60)
Test	Planned	0.00 (0.00; 1.09)	1.00 (0.37; 1.54)	2.00 (1.50–2.12)	2. (1.50; 2.14)	2.00 (1.50; 2.13)	1.80 (1.37; 2.12)	1.00 (0.00; 1.35)	0.00 (0.00; 1.09)
	6 months	0.23 (0.07; 0.79)	0.61 (0.16–1.04)	1.32 (1.01; 1.56)	1.43 (0.86; 1.77)	1.19 (0.85; 1.48)	1.02 (0.93; 1.44)	0.47 (0.14; 0.99)	0.17 (0.03; 0.75)
	12 months	0.13 (0.00; 0.67)	0.57 (0.18; 0.92)	1.13 (0.79; 1.37)	1.15 (0.77; 1.54)	1.02 (0.76; 1.45)	1.03 (0.80; 1.36)	0.31 (0.10; 0.91)	0.00 (0.00; 0.69)

The comparative analysis between the test and control groups showed no statistical difference in the amount of tissue planned to be removed and the result obtained after 6 months and 1 year. The differences between planned and removed tissue observed in the control group varied from a median of −0.09 to 0.29 mm in 6 months of follow‐up and from a median of 0.00 to 0.55 mm in 12 months. In the test group, these differences varied from −0.05 to 0.70 mm in 6 months of follow‐up and from 0.00 to 0.81 mm in 12 months (Table 3).

**TABLE 3:** Median and interquartile range of the difference of the marginal gingival position between the planning and 6‐, 12‐ months of follow‐up.

Group	Period/Tooth	14	13	12	11	21	22	23	24
Control	Planned – 6 m	0.00 (−0.17; 0.45)	0.10 (−0.12; 0.56)	0.24 (−0.38; 0.45)	0.29 (0.05; 0.58)	−0.09 (−0.37; 0.60)	0.20 (−0.07; 0.66)	0.08 (−0.14; 0.68)	−0.08 (−0.29; 0.58)
	Planned – 12 m	0.09 (−0.22; 0.53)	0.28 (0.15; 0.91)	0.45 (−0.09; 0.78)	0.55 (0.14; 1.01)	0.18 (−0.13; 0.74)	0.30 (0.13; 0.99)	0.09 (−0.13; 0.61)	0.00 (−0.02; 0.48)
	6–12 m	0.08 (−0.12; 0.17)	0.19 (0.04; 0.41)	0.37 (0.11; 0.50)*	0.09 (0.06; 0.41)	0.12 (0.04; 0.79)	0.26 (0.09; 0.45)*	0.16 (0.03; 0.28)	0.16 (−0.01; 0.29)
Test	Planned – 6 m	−0.05 (−0.15; 0.18)	0.45 (0.00; 0.64)	0.42 (0.29; 0.98)	0.62 (0.39; 0.97)	0.70 (0.49; 1.13)	0.62 (0.43; 0.94)	0.09 (−0.13; 0.61)	−0.02 (−0.15; 0.48)
	Planned – 12 m	0.00 (−0.02; 0.33)	0.38 (0.00; 0.67)	0.71 (0.35; 1.17)	0.81 (0.64; 1.03)	0.74 (0.61; 1.23)	0.64 (0.51; 0.95)	0.22 (−0.17; 0.75)	0.00 (0.00; 0.44)
	6–12 m	0.07 (0.04; 0.13)	0.07 (−0.07; 0.14)	0.11 (0.07; 0.27)	0.11 (0.05; 0.36)	0.12 (0.02; 0.17)	0.09 (0.00; 0.14)	0.11 (−0.03; 0.19)	0.05 (−0.01; 0.12)

* *p* < 0.05 More coronal of the marginal gingival position than the test group comparing the variation between the 12 with 6 m period – Maan‐Whitney test.

Furthermore, in the 12‐month STL file overlay, a coronal positional relapse of the gingival margin was observed in all groups of teeth, regardless of the surgical technique used. The lateral incisors (12 and 22) from the control technique presented more coronal positioning of the gingival margin at 12 months compared to the 6‐month follow‐up than the same teeth in the test group (*p* < 0.05). No differences were noticed between the techniques tested concerning the positioning of the gingival margin in the 6‐ or 12‐month periods in relation to the marginal tissues observed at the baseline period.

### 3.2 Postoperative Pain Analysis

The treatment of gummy smiles through guided surgery reduced the patients' perception of pain (Table 4). The level of pain presented by patients was low in both groups, with reports of minimal discomfort after the 15‐day follow‐up period.

**TABLE 4:** Median and interquartile interval of the VAS scale data observed in both groups in all experimental periods.

Period	Control	Test
3 days	0 (0,00; 3.25)	0.00 (0.00; 0.00)
7 days	0.00 (0.00; 0.50)	0.00 (0.00; 0.00)
15 days	0.00 (0.00; 0.50)	0.00 (0.00; 0.00)
30 days	0.00 (0.00; 0.00)	0.00 (0.00; 0.00)

### 3.3 Participant Satisfaction

All patients reported that they were satisfied with the surgical procedure results, with 5 patients giving the procedure a score of 9 and 15 patients giving it a score of 10. All patients reported that they would recommend the same surgical procedure to correct their gummy smile, if necessary, to friends/relatives.

## 4 Discussion

Accuracy in surgical techniques for gummy smile correction is essential for good clinical results and patient satisfaction. Millimetric differences can impact the clinical success associated with the surgical procedure to correct altered passive eruption, which makes this procedure critical [12, 17]. In the current study, the application of the guided surgery technique did not improve the predictability of the surgical treatment of altered passive eruption, showing no difference between virtual planning and freehand surgical technique in up to 1 year of follow‐up compared to the freehand surgical technique.

An important finding of this clinical study was maintaining the gingival tissue with no recession observed. On the other hand, all cases in both groups, at 6 and 12 months of follow‐up, showed some level of coronal repositioning of the gingival margin compared with the marginal gingival level planned (maximum median values of 0.70 and 0.81 mm at 6 and 12 months respectively for the test group and of 0.29 and 0.55 mm at 6 and 12 months respectively for the control group). A possible explanation for this finding is that the osteotomy performed in this clinical study to reestablish the supracrestal tissue attachment was between 2 and 3 mm so as not to cause gingival recession. However, the supracrestal tissue attachment varies between individuals and can be between 2 and 4 mm [6, 17]; therefore, the biological space reestablished may cause small recurrences after the clinical crown lengthening procedure [8] when the osteotomy performed is inferior to the patient's supracrestal tissue attachment. However, we emphasize that the coronal advancement was minimal and did not interfere with the patient's satisfaction with the surgical procedure, which states the necessity of customizing osteotomy in relation to the gingival phenotype to avoid recessions. However, more clinical studies with this specific proposition should be conducted to test this hypothesis. Another possible associated factor is unsatisfactory patient hygiene. Despite being one of the study's exclusion criteria, some participants returned with visible plaque in the upper anterior region of the maxilla and gingivitis, which may be related to gingival hyperplasia.

The main concern regarding different gingival phenotypes is gingival tissue recession. The thin phenotype is more susceptible to tissue recession. However, in our study, no difference in recession was found between the control and test groups, suggesting that the difference in gingival phenotype between groups did not impact our study results.

Regarding the accuracy of the guided surgery method compared to the conventional method in obtaining clinical crown augmentation, no statistically significant differences were observed between the methods for this purpose. In fact, a previous clinical study that evaluated altered passive eruption treatment methods using guided and conventional surgeries showed similar results to this study [14, 15]. Therefore, we believe that the advantage of the digital flow in treating gummy smiles does not seem to be in the accuracy of surgical techniques for correcting altered passive eruptions, but rather in the general context of diagnosing this condition [7, 11, 12].

Another finding in this study was the reduction of the postoperative pain associated with the guided surgery procedure. Guided surgery involved less manipulation of the flaps, which may have impacted the postoperative pain perceived by patients [11]. More conservative surgical techniques are related to lower postoperative pain [18], and one of the advantages of digital flow is the possibility of promoting less invasive surgical procedures [11, 14, 18]. However, the impossibility of blinding patients may have interfered with the pain observed in this study.

The primary outcome of the sample size calculation was clinical measurements of crown length, which is a more objective criterion for determining the superiority of one treatment over another compared to postoperative pain. Another study that compared the same techniques tested in the current study did not present postoperative pain reported by participants between the different groups [14]. It is worth noting that the number of patients in the present study who had pain events was small, and the difference found between the groups may be due to the more significant impact that patients with moderate or severe pain events have in small samples. Future studies are necessary to describe whether this type of pain reduction effect associated with guided surgery is consistent.

Although a gummy smile is a condition that has been described and treated for a long time, performing surgical procedures with the removal of soft tissue associated with the bone contour is successfully applied in the treatment of altered passive eruption; this procedure is sometimes not effective in long‐term maintenance of results or overall patient satisfaction [13, 19]. In the present study, a high level of patient satisfaction with the procedure was observed despite the more coronal positioning of the gingival margin of the lateral incisors in the control group. The lateral incisor is anatomically the tooth with the highest variation in coronal length, root, and length of biological space. This higher variability makes managing these teeth more challenging, and guided surgery could make this more predictable [20]. However, these slight morphological variations and the position of the lateral incisors were not noticeable to dentists and patients in a cross‐sectional clinical study, which is a possible explanation for the lack of differences in patient satisfaction between the different groups in this study [21].

The application of technologies such as digital smile planning [2], CT scans [14], and oral scanning [11] enhances the diagnosis of gummy smiles, making the procedure more consistent with patients' expectations [7, 22]. Using these technologies together provides the dentist with educational methods to explain to the patient what they can expect from this procedure [2]. One of the reasons for the low number of differences observed between the two surgical techniques tested in this study could be related to the experience of the clinician who performed the procedure in our study [14]. In this way, less experienced professionals can benefit from the guided surgery technique.

The results of this study should be analyzed with caution since it has some limitations. This study did not consider the patient's periodontal health after the procedure. This is relevant since gingival inflammation may contribute to gingival hyperplasia, potentially affecting recurrence. Additionally, the surgical time required for the two techniques should have been considered. This can influence post‐operative pain, which is not only associated with the degree of invasiveness of an intervention but also with the total time of the procedure. This clinical trial is not blinded and has a short follow‐up period (12 months). In this way, new clinical trials with larger sample sizes and longer follow‐ups to assess long‐term stability and patient‐centered outcomes (e.g., aesthetic satisfaction and quality of life) are indicated. Despite the limitations, this study can give directions to the clinician regarding the value of both techniques.

## 5 Conclusions

With caution, due to the limitations of this study, it can be concluded that both the use of surgical guides and the conventional technique are effective for treating altered passive eruption with a high level of patient satisfaction, with a slight difference when evaluating lateral incisors that are not noticeable to patients.

## Conflicts of Interest

The authors declare no conflicts of interest.

## Data Availability

The data that support the findings of this study are available from the corresponding author upon reasonable request.
